# Structure of Patt1 human proapoptotic histone acetyltransferase

**DOI:** 10.1007/s00894-013-2043-1

**Published:** 2013-11-19

**Authors:** Roch Paweł Jędrzejewski, Rajmund Kaźmierkiewicz

**Affiliations:** Laboratory of Biomolecular Systems Simulations, Intercollegiate Faculty of Biotechnology, University of Gdańsk and Medical University of Gdańsk, Kładki 24, 80-822 Gdańsk, Poland

**Keywords:** GNAT family proteins, HAT histone acetyltransferases, Homology modeling, Molecular dynamics simulation, Protein structure prediction

## Abstract

The results of modeling of a novel human histone acetyltransferase Patt1 are presented here. This protein belongs to the GNAT GCN5 family and shows proapoptotic activity in human hepatocellular carcinoma cells. Patt1 is an attractive therapeutic target. The sequence analysis, fold recognition predictions and homology modeling of Patt1 protein structure were performed. N- and C- termini of Patt1 were unstructured. Central part revealed classical GNAT fold–central 7-stranded beta sheet core surrounded by intervening 4 alpha helices. The model was assessed with the methods for protein structure validation PROQ and MetaMQAPII. The all-atom 12 ns molecular dynamics simulation of Patt1 model with TIP3P water model and counterions was conducted. All assessment methods implemented resulted in conviction that the model was of quality that could provide confident structural information to infer sequence-structure-function relationships of Patt1. Phe186 and Cys137 were identified as residues engaged in acetyltransfer reaction and the clues for the identification of reaction mechanism were proposed. The knowledge of detailed molecular architecture of Patt1 is not only the key to understanding its mechanistic functional properties but it also opens the possibility of rational drug and protein design experiments, leading to development of effective therapeutic methods.

## Introduction

In eukaryotic cells the acetylation of histone N-terminal tails is the key regulatory mechanism in histone code and the execution of epigenetic information [1–3]. The information encoded in modification patterns of N-terminal histone tails is crucial for chromatin remodeling. The modification of tails is then read out by specific proteins by means of molecular recognition. Chromatin remodeling machines (CRM) complexes take the input information encoded in the form of the modifications order. This drives the chromatin remodeling processes and opens access to chromatin. These events transfer chromatin to transcriptionally active state in which DNA template is accessible for transcription factors and other regulatory proteins that bind DNA. This results in expression of specific genes. Switching chromatin to an open state is induced by acetylation [1–3]. Acetylation is performed by vast array of enzymes from various families [4]. Many different families of proteins are engaged in histone acetylation [5, 6]. Histone acetyltransferases (HAT) perform acetylation of N-terminal histone tails at specific lysines. Dysfunction of histone acetyltransferases leads to carcinogenic processes [7, 8]. This aspect of acetyltransferases has gained growing attention in recent years. The GNAT family of acetyltransferases [9–11] contains the first discovered proteins that possess histone acetylation activity that was connected with gene activation [12]. A recently discovered member of this group Patt1 (protein acetyltransferase-1) has been shown to acetylate histone H4 in vitro and in vivo [13]. Activity of this protein was linked to promotion of apoptosis in human hepatocellular carcinoma cells. There is no information available about the structure of this new human histone acetyltransferase. Patt1 protein all-atom tertiary structure was modeled with the use of theoretical methods. All structure assessment methods and the results of molecular dynamics simulation indicated that the model is correct. The model exhibits all features of GNAT canonical topology: the core of protein is formed by central beta sheet composed of seven strands. Alpha helices are mixed with beta sheets and surround the structure on both sides. The enzyme active site is located at the edge of beta sheet, between β5 and β6 structural elements. Amino acid residues engaged in acetyltransfer reaction were identified: the aromatic rings of Phe185, Phe186, and Phe192 form a stacking system. Their role is alternative to general acid catalysis. The spatial localization of Cys137 is similar to analogous cysteine residue in Esa1 histone acetyltransferase where acetyltransfer reaction proceeds through an acetyl-cysteine enzyme intermediate. The model represents the first structural data regarding Patt1 protein.

## Materials and methods

### Patt1 sequence analysis

The searches of an nr National Center for Biotechnology Information (NCBI) sequence database [14] were performed using a PSI-BLAST algorithm [15] with Patt1 sequence [13] as an input (query). Default parameters were used, except for the “max target sequences” which was set to 1000. After eight iterations the set of resulting sequences showing statistically significant similarity was collected and used to construct the multiple sequence alignment (MSA) of Patt1 family. MSA was computed with the use of MUSCLE program [16, 17]. CDS (conserved domain search) [18] was used for assignment of the sequence to specific protein superfamily. Manipulation of sequence data was done with use of BioEdit [19] and SeaView [20] programs. ClustalX [21] was used to build phylogenetic trees (data not shown). TreeView [22] program was used to visualize them.

### Fold recognition analysis

Fold recognition methods are based on detection of compatibility of a given protein sequence with sequence profiles and/or structures of proteins deposited in structural databases. In order to identify a suitable template for homology modeling of Patt1 structure the MSA of Patt1 family was submitted to GeneSilico Metaserver [23], which is a gateway to >30 third party methods of automated protein secondary structure prediction, solvation and disorder prediction (reference: https://genesilico.pl/meta2/).

### Patt1 structure modeling

The MODELLER program [24] available through GeneSilico Web Toolkit (https://genesilico.pl/toolkit/modeling) was used to construct an all-atom tertiary model of Patt1. Swiss-Pdb Viewer (SPDBV) program [25] was used for structure visualization and manipulation.

### Model quality assessment

All theoretical models must be thoroughly evaluated before using them as source of information of molecular architecture and composition of key structural elements. The state-of-the-art methods of protein structure quality assessment specially designed to score theoretical models were used. Among them were MetaMQAPII [26] and ProQ [27] methods implemented in GeneSilico Web Toolkit (https://genesilico.pl/toolkit/).

The ProQ web server [28] (available at Stockholm Bioinformatics Center website: http://www.sbc.su.se/∼bjornw/ProQ/ProQ.html) was also used.

### Energy minimization and molecular dynamics simulations

Molecular dynamics simulation and energy minimization were carried out using AMBER9 package [29]. Initial energy minimization in vacuo with unrestrained system was performed. Steepest descent algorithm was used for the first 6000 steps and conjugate gradient algorithm for following 24,000 steps. Cutoff value for treating long-range electrostatic interactions was 12.0. Simulation was performed with explicit solvent water model TIP3P. Counterions were added to neutralize net charge on protein surface. Long-range electrostatic interactions were evaluated using particle mesh Ewald summation method with periodic boundary conditions. Langevin thermostat method was used to control temperature of the system. The temperature was set to 300 K. The simulation duration was 12 ns, with time step (dt) of 2 fs.

## Results and discussion

### Patt1 sequence analysis

**Fig. 1:**
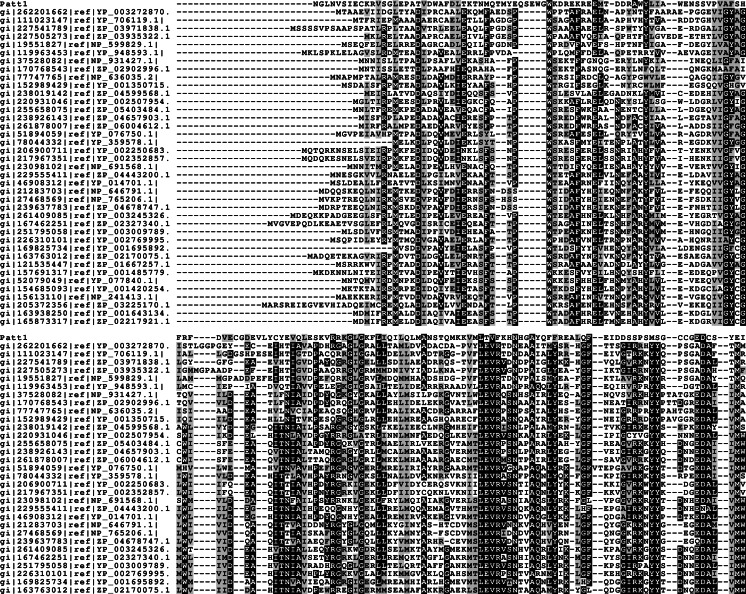
Multiple sequence alignment of Patt1 amino acid sequence (residues 50–237) and representative sequences of significant similarity to Patt1. GNAT family conserved sequence motifs [11] are discernible

The sequence of Patt1 protein is 237 amino acid residues long. The set of sequences resulting from preliminary PSI-BLAST (default parameters, except for the “max target sequences = 1000” setting) search contained various members of GNAT family acetyltransferases from all domains of life. According to CDS results the region of similarity to GNAT superfamily was detected between approximately 112–187 amino acid residues. MSA was constructed and submitted to GeneSilico Metaserver. Disorder prediction indicated that the N-terminal (1–50 amino acid residues) and C-terminal regions (215–237 amino acid residues) of Patt1 protein were unstructured. The predicted regions of alpha helices and beta sheets conformed to the (α)-β-α-α-β-β-β-α-β-α-β-β pattern, which is in agreement with the canonical GNAT fold topology (Fig. 1). The prediction of first alpha helix is uncertain since it occurs in the region of predicted disorder and is likely to represent “intrinsically disordered” locally stable alpha helix in the lack of defined tertiary structure.

### Fold recognition and Patt1 structure modeling

Fold recognition methods implemented in GeneSilico Metaserver were used to search within PDB database for protein structures solved by X-ray crystallography and NMR. The structure scoring by fold recognition servers provides means of selection of suitable template structures. Fold recognition servers returned many structures that can be used as possible templates in homology modeling. The templates were in most cases members of a newly discovered GNAT subfamily of pita-like proteins. The choice was based on sequence similarity and structure scoring by fold recognition servers. The structure of RimI [30] was chosen as a modeling template. The Patt1 model was constructed with use of the MODELLER program.

### Patt1 model assessment

The Patt1 model was scored by ProQ and MetaMQAPII methods. The ProQ LGscore: 2.082 and MaxSub: 0.275 indicated that the model was of sufficient quality “fairly good model”, and can be used in subsequent stages of analysis. MetaMQAPII confirmed correctness of the model.

### Patt1 molecular dynamics

**Fig. 2:**
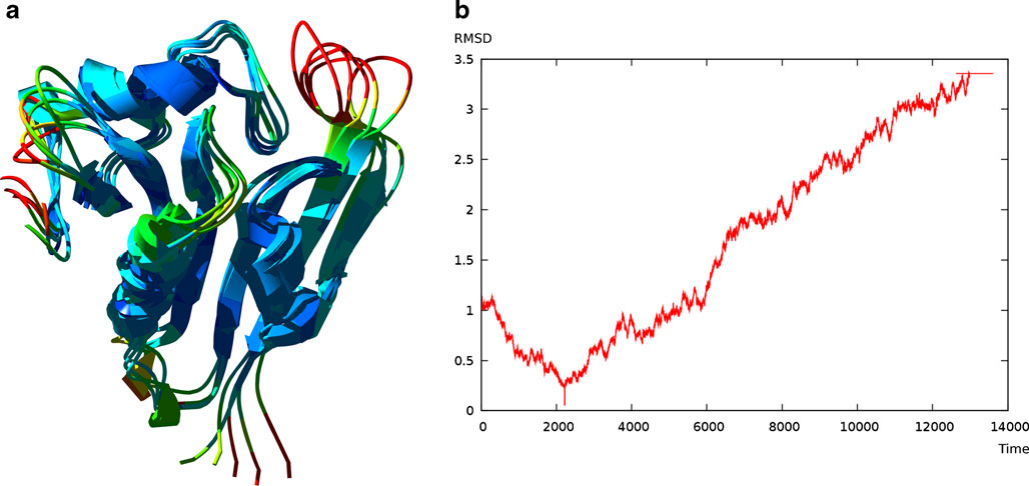
**a** Ensemble of structures from MD simulation representing stages of the model after 4, 6, 8, 10 and 12 ns. Colors corresponds to RMSD values. **b** Plot of RMSD value averaged for C-alpha atoms over time

The binding site of the peptide and AcCoA is located at the catalytic center of the enzyme, surrounded by B-sheet elements β6 and β7. The extended loop between these structural elements showed highest conformational flexibility during simulation. This behavior corresponds with available literature data for RimI where β6-β7 loop movements correlate with acetyltransfer reaction. The protein structure was stable during simulation, no unfolding of tertiary structure was observed (Fig. 2).

### Patt1 structure analysis

**Fig. 3:**
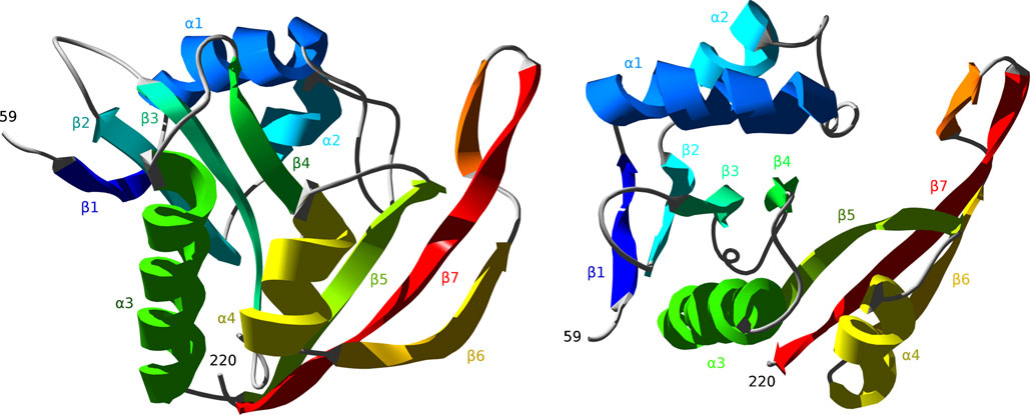
Overview of the Patt1 structure. Schematic ribbon representation featuring sheets and helices. The second picture is rotated by 90° along x axis into image plane, giving impression of “top view” over the edge of the central β-sheet. The β-like structural element located between β6-β7 sheets is unstable and dissolves during MD simulation into extended loop

**Fig. 4:**
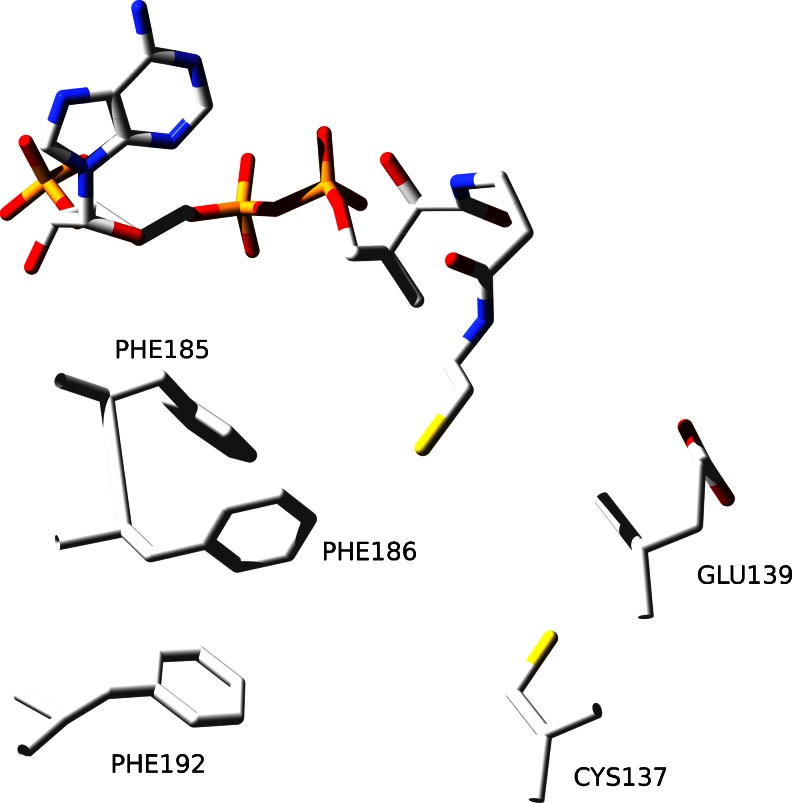
Spatial localization of key residues in Patt1 active site. The CoA ligand was copied from RimI structure

The region that comprises GNAT fold domain is located between 59 and 220 amino acid residue. Patt1 reveals classical GNAT fold with the central region of beta sheet surrounded by alpha helices. The sequence of Patt1 structural elements is: β1-α1-α2-β2-β3-β4-α3-β5-α4-β6-β7 (Fig. 3). In the core region, the enzyme active site is located at the edge of the central beta sheet, surrounded by α3 and α4 helices in the region of Acetyl-CoA binding. The β6-β7 sheet structural elements along with the loop between α1 and α2 helices enclose the active site in the region of acetylated peptide binding. The characteristic feature of GNAT family proteins, the V-like splay shape between the β4-β5 (the specific region of active site where acetyltransfer reaction takes place) structural elements is also present in the Patt1 model. The high quality of the model allows detailed predictions about the specific amino acid residues function to be made. The sequence region “RRKGLG” spanning between 147 and 152 amino acid residues in Patt1 sequence was shown to be the GNAT signature motif Arg/Gln-X-X-Gly-X-Gly/Ala responsible for recognition and binding of CoA [13]. In the model it is located in the loop between β4-α3 and at the beginning of α3 helix. Particularly interesting are other interactions with CoA in Patt1 structure. In the α4 helix there are two highly conserved phenylalanine residues present: Phe185 and Phe186 that are directed to CoA substrate. With the next highly conserved Phe192 residue located within α4-β6 loop that follows α4 helix they form a system of stacking rings. CoA adenine rings are trapped in contacts with the plane of the Phe185 ring. These interactions contribute to proper positioning of the ligand. Structural alignment of Patt1 and RimI reveals that spatial localization of Phe186 residue in Patt1 corresponds to Tyr115 in RimI structure. Tyr115 (RimI) has been assigned a role of the active site acid [30]. The interactions with stacking rings systems have been shown to act as an alternative to general acid catalysis [31]. Therefore, it is proposed that this spatial arrangement of conserved phenolic rings should take part in acetyltransfer reaction (Fig. 4). In the structure of RimI the amide backbone of the residue Ile69 has a role of stabilizer of polarization of acetyl group in the process in which the tetrahedral intermediate is formed during acetyltransfer reaction. This position is occupied by a conserved Val140 residue in Patt1. In the structure of Patt1 in the active site there is conserved Cysteine 137 residue located with sulfhydryl group directed to sulfhydryl group of CoA (Fig. 4). The presence of cysteine in the active site might suggest its involvement in reaction mechanism. Structural alignment of Patt1 model with yeast Esa1 structure [32] was performed. Esa1 is a histone acetyltransferase that belongs to the MYST subfamily. In Esa1 the strictly conserved Cys304 residue is responsible for the common acetylation reaction mechanism (that proceeds through an acetyl-cysteine enzyme intermediate) [33]. Both Patt1 and Esa1 perform acetylation of histone H4. The structural alignment reveals that both Patt1 Cys137 and Esa1 Cys304 residues are located in the active site, at the beginning of β4 sheet. The Glu139 residue is localized in close proximity in the Patt1 model (within the β4 sheet structural element) which has been assigned a role as an active site base [13]. Conserved residues are located in the region of specific interactions with the peptide that undergoes acetylation. These residues surround the peptide substrate and position it prior to acetyltransfer. Among them there are residues located in the α1-α2 loop. This loop is extended in comparison with RimI structure, due to probable insertion event. The most highly conserved residue in this region–Trp92 is engaged in stacking interactions with conserved Tyr85. This specific positioning increases the strength of hydrogen bonding interactions made by Tyr85 at the recognition interface of Patt1. The conserved Glu86 residue is directed into the space of peptide binding. In the loop that extends between β6-β7 sheet elements a sequence ‘GCCG’ (Gly203, Cys204, Cys205, Gly206) is located. Glycine residues facilitate conformational flexibility in this region. The presence of hydrophobic amino acid residue in the loop that is exposed to solvent indicates that the loop constitutes a part of recognition interface. In the structure of RimI in this region there are strictly conserved Tyr129 and Tyr130 residues that make contacts with the ligand peptide backbone [30]. Movements of these residues in RimI structure accompanies critical steps of acetyltransfer reaction. Met81(Patt1) is at the place of His23(RimI). Its proposed function, as in the case of RimI analog is to increase affinity to CoA by hydrophobic interactions.

## Conclusions

The all-atom tertiary structure of a novel human histone acetyltransferase Patt1 was modeled by means of theoretical methods. The model was assessed with the state-of-the-art methods for protein structure validation, and subjected to 12 ns molecular dynamics simulation. The integrity of structure was retained. The model allowed to infer sequence-structure-function relationships of Patt1. The key residues identified as involved in acetyltransefer reaction were: Phe185, Phe186, Phe192, and Cys137. Patt1 promotes apoptosis in human hepatocellular carcinoma cell lines. The availability of effective therapy is strongly dependent on structural data of proteins. Three-dimensional molecular structure can be used as a map in performing protein design experiments and evaluation of their results. It also opens the possibility of rational drug design and can help in the development of personalized medicine.
